# The Association Between Serum Ergothioneine Concentration and Japanese Dietary Habits: The Third Survey of the ROAD Study

**DOI:** 10.3390/nu17030517

**Published:** 2025-01-30

**Authors:** Kosuke Suzuki, Yoshihisa Kaneda, Takayuki Izumo, Yoshihiro Nakao, Toshiko Iidaka, Chiaki Horii, Shigeyuki Muraki, Hiroyuki Oka, Hiroshi Kawaguchi, Toru Akune, Hiroshi Hashizume, Hiroshi Yamada, Kozo Nakamura, Sakae Tanaka, Noriko Yoshimura

**Affiliations:** 1 Department of Prevention Medicine for Locomotive Organ Disorders, 22nd Century Medical and Research Center, The University of Tokyo, Tokyo 113-8655, Japan; kosule_suzuki@suntory.co.jp (K.S.);; 2 Institute for Science of Life, Suntory Wellness Limited, 8-1-1 Seikadai, Seika-cho, Soraku-gun, Kyoto 619-0284, Japan; 3 Department of Orthopaedic Surgery, Sensory and Motor System Medicine, Graduate School of Medicine, The University of Tokyo, Tokyo 113-8655, Japan; 4 Division of Musculoskeletal AI System Development, Faculty of Medicine, The University of Tokyo, Tokyo 113-8655, Japan; 5 Nadogaya Hospital, Chiba 277-0084, Japan; 6 National Rehabilitation Center for Persons with Disabilities, Saitama 359-0042, Japan; 7 Department of Orthopedic Surgery, Wakayama Medical University School of Medicine, Kimiidera 811-1, Wakayama 641-8509, Japan; 8 Towa Hospital, Tokyo 120-0003, Japan

**Keywords:** ergothioneine, ROAD study, dietary habit, fish

## Abstract

**Background/Objectives:** As a result of aging societies, the increasing number of older adults requiring nursing care has become a serious issue and the extension of healthy life expectancy has become an urgent priority. Ergothioneine (EGT) is a sulfur-containing amino acid found in foods such as mushrooms. Low EGT blood concentrations have been reported to be associated with the risk of onset and progression of various diseases. However, the distribution of EGT blood concentrations and their association with dietary habits in the Japanese general population remains unclear. **Methods:** This cross-sectional study was conducted using data from the third survey of the Research on Osteoarthritis/osteoporosis Against Disability (ROAD) study, which analyzed 1457 participants (474 men and 983 women) aged ≥ 40 years. Serum EGT concentrations and their association with dietary habits were analyzed. **Results:** Serum EGT concentrations (1) peaked in the 70s in men and the 60s in women, (2) were higher in women than in men, and (3) showed a significant positive correlation with fish intake and nutrients commonly found in fish. **Conclusions:** In the present study, we report for the first time an age- and sex-specific serum EGT distribution in a Japanese population and its association with dietary habits, particularly fish intake. These findings help define normal and abnormal EGT levels and suggest new potential sources of EGT.

## 1. Introduction

The rapid medical advancements achieved in the latter half of the twentieth century have extended the average life expectancy of the population. According to future population predictions published by the Japanese government in 2017, the average life expectancy in 2040 is forecasted to be 83.3 years for men and 89.6 years for women [1]. However, this drastic increase in life expectancy has also led to an increase in the number of older adults with anxiety and feelings of isolation who require long-term care. Therefore, it has become an urgent priority to extend not only life expectancy, but also healthy life expectancy, and daily dietary nutrition plays a crucial role in achieving this.

Ergothioneine (EGT) is a sulfur-containing amino acid found abundantly in mushrooms that is known for its potent antioxidant and anti-inflammatory functions [2]. EGT is a substrate of the carnitine/organic cation transporter (OCTN1/SLC22A4) and is widely expressed in various organs, including the brain and intestines. It is efficiently transported across the blood–brain barrier following oral ingestion [3,4]. EGT is also known as a “longevity vitamin” [5] and known to be associated with a variety of biological functions. Irwin et al. [6] reported that low EGT levels were associated with a decline in cognitive function. Additionally, low levels of blood EGT concentrations have been reported to be associated with the onset and progression of Parkinson’s disease [7], frailty [8], a decreased walking speed [9], chronic kidney disease [10], Crohn’s disease [11], and sickle cell disease [12]. Einar et al. [13] also reported that high levels of blood EGT concentrations were associated with decreases in cardiovascular and overall mortality. Furthermore, Katube et al. [14] found that EGT intake for 4 weeks improved sleep quality in individuals with high levels of anxiety and sleep complaints. In addition, in mice, EGT administration extended life expectancy, improved age-related frailty [15], and increased endurance and muscle synthesis when combined with training [16]. These previous findings suggest that EGT levels in the blood may be a promising predictive or protective biomarker for various diseases.

However, to our knowledge, no studies have been reported on the distribution of EGT levels among the general Japanese population that could help define normal or abnormal levels. Additionally, dietary intake is the primary source of EGT because it cannot be synthesized by humans. Among all foods, mushrooms have been reported to be the richest source of EGT [17], but data on EGT content in foods other than mushrooms are limited, and the relationship between dietary intake and blood EGT levels in Japan remains unknown.

Since 2005, we have conducted the Research on Osteoarthritis/osteoporosis Against Disability (ROAD) study, a cohort study of community-dwelling residents, and reported on epidemiological indicators and risk factors including nutrition for musculoskeletal diseases such as osteoarthritis, osteoporosis, sarcopenia, and lumbar spondylosis, as well as lifestyle-related diseases such as metabolic syndrome [18,19,20,21,22,23,24,25]. In the present study, using the results of the third survey of the ROAD study, we aimed to clarify the distribution of EGT blood concentrations and their association with dietary habits in the Japanese general population.

## 2. Materials and Methods

### 2.1. Participants

The ROAD study, which started in 2005, is a nationwide prospective study in Japan comprising population-based cohorts of three communities: an urban area (Itabashi, Tokyo), a mountainous area (Hidakagawa, Wakayama), and a coastal area (Taiji, Wakayama). Detailed recruitment methods for the ROAD study have been described previously [18,19]. To date, we have created a baseline database including clinical and genetic information from 3040 inhabitants (1061 men and 1979 women; age range, 23–95 years) recruited from the resident registration lists of the three communities mentioned above.

The third survey of the ROAD study, which serves as the baseline of the present study, was conducted in 2012–2013. Invitation letters were sent to individuals who had taken part in the previous two ROAD study surveys. In addition to former participants, inhabitants aged ≥40 years who were willing to join the ROAD study were also asked to join. The recruitment of new participants was conducted through local government publications, with no restrictions other than having to be at least 40 years of age. Throughout the study, the inclusion criteria were set as follows: (1) the ability to walk to the clinic where the survey was performed, (2) the ability to provide self-reported data, and (3) the ability to understand and sign an informed consent form. There were no specific exclusion criteria.

A total of 2566 (837 men and 1729 women; urban area [n = 845], mountainous area [n = 769], coastal area [n = 952]) residents participated in the third visit (EGT baseline). Among 1721 participants in the EGT baseline in the mountainous and coastal areas, we used data from 1457 (474 men and 983 women) whose serum samples could be measured for EGT. Data from urban residents were not included in the EGT baseline because no serum measurements were performed in that cohort.

### 2.2. Examination at EGT Baseline (Third Survey of ROAD Study)

#### 2.2.1. Questionnaire, Interviews, and Anthropometric Measurements

Measurement data were collected from the participants in the third survey of the ROAD study. At the third examination, participants completed a 200-item questionnaire administered by an interviewer that included lifestyle-related information such as primary occupation, smoking habit, alcohol consumption, physical activity level, and medical history, including history of prescription medications. Anthropometric measurements, including height (m), weight (kg), body mass index (BMI; calculated as weight [kg]/height [m^2^]), and hand grip strength (kg), were also taken.

#### 2.2.2. Dietary Assessment

The participants completed a self-administered brief diet history questionnaire (BDHQ), which was developed as a short version of a validated self-administered diet history questionnaire focused on the typical Japanese diet [26] that has been widely used for dietary assessments in Japan [25,27,28] at home. The responses were then reviewed by well-trained interviewers at the clinic to ensure that all question items had been answered. The BDHQ assesses the frequency of intake of 56 food and beverage items over the past month and calculates the daily intake of energy and selected nutrients using a specific computer algorithm [29]. In the present study, the intake levels of total energy and 46 key nutrient factors were analyzed.

#### 2.2.3. Measurement of Serum EGT Concentrations

Commercially available isotope-labeled EGT-d9 (Toronto Research Chemicals, Toronto, ON, Canada) was used as the internal standard (IS). For serum EGT samples, 600 μL of acetonitrile was added to a mixture containing 30 μL of serum, 30 μL of extra pure water, and 10 μL of a 10 μM IS solution. Then, the mixture was vortexed and centrifuged at 10,000× *g* at 4 °C for 5 min. Subsequently, 100 μL of the supernatant was collected and diluted with 100 μL of mobile phase A (water/formic acid, 1000:1, *v*/*v*). Next, 5 μL of the samples were injected and analyzed using ultra-high-performance liquid chromatography–tandem mass spectrometry (LC-20AD System; Shimazu, Kyoto, Japan) coupled to a quadrupole tandem mass spectrometry instrument (API4000; AB Sciex, Tokyo, Japan). Chromatographic separation of EGT was performed using a ZIC-cHILIC (150 × 2.1 mm, 3 μm; 100 Å, Merck Millipore Corporation, Burlington, MA, USA) column with mobile phase B (acetonitrile/formic acid, 1000:1, *v*/*v*). Gradient elution was performed as follows: 0–10 min, 5% A/95% B to 80% A/20% B; 10–11 min, 80% A/20% B; 11–11.1 min, 80% A/20% B to 5% A/95% B; and 11.1–15 min, 5% A/95% B at a flow rate of 0.4 mL/min. The mass spectrometer was operated in multiple reaction monitoring modes with positive electrospray ionization EGT and EGT-d9 mass transitions of *m*/*z* 230.0 > 186.4 and *m*/*z* 239.0 > 195.0, respectively. The curtain gas, collision gas, temperature, Gas1, Gas2, and declustering potential were set at 30 psi, 5 psi, 400 °C, 30 psi, 30 psi, and 50 V, respectively. The concentration of the samples was determined by weighted (1/×2) least square linear regression on the peak area ratio (EGT/IS) using a calibration curve that included concentrations of 0.1, 0.2, 0.5, 1, 2, 5, and 10 μM. Samples with concentrations exceeding the maximum standard concentration were diluted and remeasured.

### 2.3. Ethical Approval

This study was approved by the ethics committees of the University of Tokyo (Nos. 1264) and carried out in accordance with the Declaration of Helsinki. All participants provided written informed consent before the study began.

### 2.4. Statistical Analysis

All statistical analyses were performed using JMP software (version 18.0; SAS Institute Inc., Cary, NC, USA). Differences in characteristics between men and women were compared using Student’s *t*-test and Dunnett’s test for each age stratum. Pearson’s correlation analysis and partial correlation analysis were used to examine the associations between serum EGT concentrations and diet and dietary nutrients.

## 3. Results

### 3.1. Characteristics of Participants

**Figure 1:**
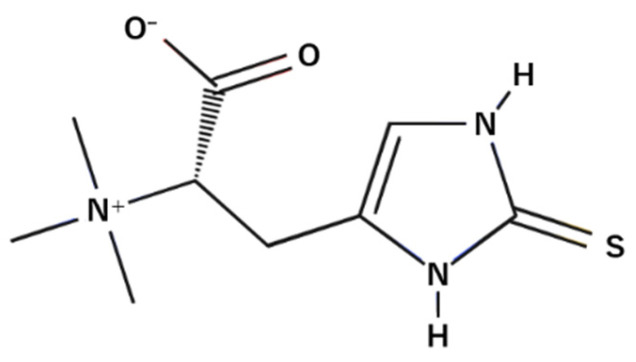
The structure of EGT (IUPAC; (2S)-3-(2-sulfanylidene-1,3-dihydroimidazol-4-yl)-2-(trimethylazaniumyl)propanoatee).

We show the EGT structure in Figure 1. Table 1 shows the background and EGT baseline characteristics of the participants. No significant difference in age was seen between men and women. On the other hand, height (*p* < 0.001), weight (*p* < 0.001), and BMI (*p* < 0.001) were significantly higher in men than in women.

### 3.2. Serum Values of EGT

**Figure 2:**
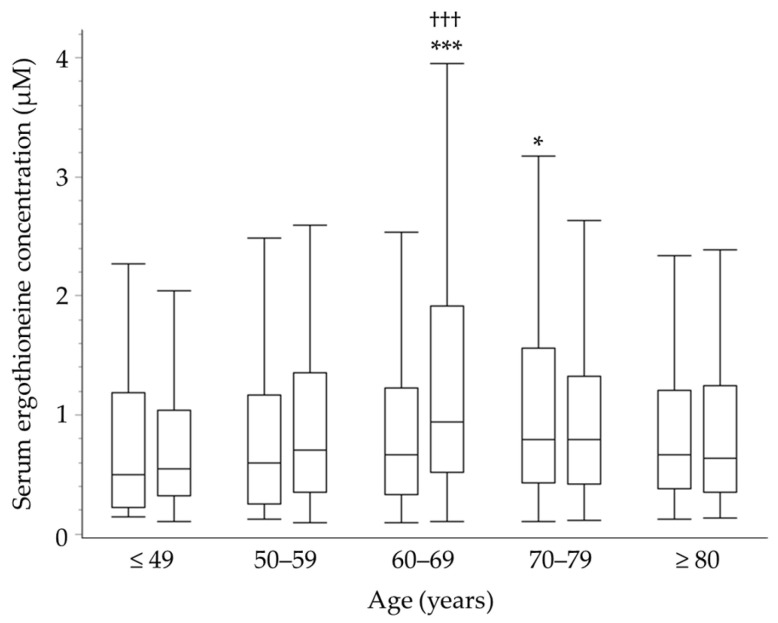
Serum ergothioneine (EGT) concentrations in each age stratum. Data are expressed as boxplots (left box: men; right box; women, for each age stratum). Dunnett’s test was used to compare between age strata (50–59, 60–69, 70–79, and ≥ 80 years) vs. ≤ 49 years by sex. *** *p* < 0.001, * *p* < 0.05. Nonpaired Student’s *t*-test was used to compare between sex by age strata. ^†††^ *p* < 0.001.

The mean values of serum EGT concentrations were significantly higher in women than in men (*p* < 0.01). Figure 2 shows the distribution of serum EGT concentrations by age and sex. The highest concentration in men was observed in the 70s age group (*p* < 0.05 vs. ≤49 years), while the peak concentration in women was in the 60s age group (*p* < 0.001 vs. ≤49 years).

### 3.3. Association Between Serum EGT Concentration and Diet History

Table 2 shows the correlation between serum EGT concentrations and the intake of various foods. Foods that showed positive correlations with EGT included total fish (*r* = 0.215, *p* < 0.001), mushrooms (*r* = 0.202, *p* < 0.001), grilled fish (*r* = 0.200, *p* < 0.001), cooked sugar (*r* = 0.173, *p* < 0.001), fish with bones (*r* = 0.168, *p* < 0.001), cooked salt (*r* = 0.157, *p* < 0.001), oily fish (*r* = 0.152, *p* < 0.001), tofu and fried tofu (*r* = 0.148, *p* < 0.001), braised fish (*r* = 0.130, *p* < 0.001), braised food (*r* = 0.128, *p* < 0.001), seaweed (*r* = 0.123, *p* < 0.001), green leafy vegetables (*r* = 0.121, *p* < 0.001), dried fish (*r* = 0.118, *p* < 0.001), carrots and pumpkins (*r* = 0.091, *p* = 0.001), cabbage (*r* = 0.075, *p* = 0.004), sake (*r* = 0.068, *p* = 0.009), radish/tuber (*r* = 0.062, *p* = 0.019), and beer (*r* = 0.054, *p* = 0.039).

On the other hand, foods that showed negative correlations included fried food (*r* = −0.132, *p* < 0.001), bread (*r* = −0.123, *p* < 0.001), ramen noodles (*r* = −0.091, *p* = 0.001), grilled meat (*r* = −0.088, *p* = 0.001), cooking oil (*r* = −0.078, *p* = 0.003), soy sauce (*r* = −0.067, *p* = 0.010), persimmon/strawberry (*r* = −0.066, *p* = 0.012), sugar (*r* = −0.064, *p* = 0.014), ham (*r* = −0.064, *p* = 0.014), hamburger steak (*r* = −0.060, *p* = 0.023), and citrus fruits (*r* = −0.056, *p* = 0.032).

### 3.4. Association Between Serum EGT Concentration and Dietary Nutrient History

Table 3 shows the correlations between serum EGT concentrations and the intake of various nutrients. Energy intake was not significantly correlated with EGT (*r* = 0.041, *p* = 0.12), whereas protein (*r* = 0.109, *p* < 0.001), animal protein (*r* = 0.121, *p* < 0.001), and animal fat (*r* = 0.070, *p* = 0.007) were positively correlated. Thirty nutrients, including minerals, vitamins, fatty acids, fiber, and carotenoids, were positively correlated with EGT, whereas vegetable lipids showed a negative correlation. Vitamin D (*r* = 0.180, *p* < 0.001), vitamin B_12_ (*r* = 0.155, *p* < 0.001), and fatty acids (n-3) (*r* = 0.123, *p* < 0.001), which are abundant in fish, showed significant positive correlations.

## 4. Discussion

In the present study, we used data from the third survey of the ROAD cohort study, which was conducted between 2012 and 2013, to reveal for the first time the distribution of serum EGT concentrations and their associations with food and nutrient intake in the Japanese population.

The results indicated that the peak age for serum EGT concentrations was in the 70s for men and the 60s for women (Figure 2). Previous reports have suggested that blood EGT levels decrease after reaching 60 years of age [6]. While this trend was consistent in women, our study identified a peak in the 70s for men. Additionally, the EGT concentration in participants aged< 40 years was lower than those in participants aged in their 60s and 70s. This result persisted even after adjusting for mushroom intake, with statistical significance for women and a trend toward significance for men, suggesting the involvement of factors other than diet (Table S1). It has been reported that oxidative stress increases with aging [30]. We hypothesize that during middle age, the defense mechanisms against increased oxidative stress lead to an upregulation of OCTN-1, resulting in elevated serum EGT levels. In old age, however, further increases in oxidative stress likely result in the consumption of EGT, leading to its decline. Nonetheless, there remain uncertainties regarding this mechanism, which needs further investigation. Furthermore, serum EGT concentrations were significantly higher in women than in men. On the other hand, a cohort study in Australia reported no such gender difference [31]. The gender differences in serum EGT levels persist even after adjusting for mushroom and fish intake (Table S2), suggesting that other lifestyle factors or genetic backgrounds may be influencing these differences. Regarding the discrepancy with the Australian study, which did not observe gender differences, further investigation is needed to determine whether this is due to population bias, genetic factors, or cultural backgrounds.

Next, we examined the relationship between serum EGT concentrations and food intake, and found a positive correlation between EGT concentrations and mushroom consumption (Table 1). Irwin et al. [6] reported a similar positive correlation between EGT concentrations and mushroom intake in a cohort study of Singaporeans, and our findings support their results. However, our results also indicated a significant positive correlation between the intake of fish, such as grilled fish, fish with bones, oily fish, braised fish, and dried fish, and serum EGT concentrations. Moreover, the association between total fish intake and serum EGT levels was stronger than that for mushrooms (fish: *r* = 0.215; mushrooms: *r* = 0.202; *p* < 0.001). Even in partial correlation analyses, fish intake showed a stronger association with serum EGT than did mushroom intake (fish: *r* = 0.168; mushrooms: *r* = 0.150; *p* < 0.001) (Table S3), suggesting that fish is independently associated with serum EGT levels. To date, EGT has been reported in mushrooms and meats [32], but to our knowledge, there have been no previous reports of its content in fish.

Furthermore, we found a positive correlation between serum EGT concentrations and nutrients such as docosahexaenoic acid (*r* = 0.175, *p* < 0.001), eicosapentaenoic acid (*r* = 0.182, *p* < 0.001), and vitamin D (*r* = 0.180, *p* < 0.001), which are abundant in fish. Although EGT is found in both plants and animals, its biosynthesis has only been confirmed in bacteria and fungi [33]. The low levels of EGT typically found in plants are thought to be acquired from soil fungi or bacteria through the roots as part of mycorrhizal symbiosis [34], while EGT in meat is thought to come from animals ingesting plants. Similarly, fish may accumulate EGT from microalgae. In fact, cyanobacteria, a type of plankton widely present in the ocean, are known to produce EGT [35,36]. Similarly, Tamara et al. [37] reported that tuna and salmon contain EGT (mean concentrations of 3.5 ± 0.2 μg/g and 3.3 ± 0.3 μg/g, respectively). EGT content in mushrooms commonly consumed in Japan is 0.056 mg/g wet mass in shimeji, 0.123 mg/g wet mass in shiitake, and 0.151 mg/g wet mass in enoki [32], values which are 10 to 30 times higher than those in tuna and salmon. However, the total fish intake is 145.3 ± 101.7 g/day, whereas mushroom intake is 10.6 ± 9.5 g/day (Table 2), which is approximately 14 times lower than fish intake. In addition, the accumulation of EGT in fish may depend on the expression levels of OCTN1 and their feed, which makes it necessary to consider the type of fish and habitats. In Japan, tuna is often consumed raw; however, in the present study, no association was found between raw fish intake and serum EGT levels (*r* = −0.040, *p* = 0.125). Further investigation of EGT content is needed in the fish commonly consumed in Japan (e.g., mackerel, horse mackerel, Pacific saury, yellowtail).

This study has several limitations. First, due to the cross-sectional nature of the analysis, it was not possible to verify a causal relationship between dietary intake and serum EGT concentrations. However, as follow-up surveys are being conducted in this cohort, it will be possible to clarify changes in EGT concentrations and the relationship between dietary intake and serum EGT levels over time.

Second, although many residents participated in the ROAD study, the participants may not fully represent the Japanese population. When comparing the baseline data of the study participants with the general Japanese population based on the 2018 National Health and Nutrition Survey, a significant difference in mean BMI was observed among participants aged ≥ 60 years (22.7 [3.4] vs. 23.4 [0.6] kg/m^2^, *p* < 0.001). Additionally, the proportion of current smokers and drinkers was significantly lower in both men and women in this study compared with the general Japanese population (smokers: men, 16.5% vs. 22.3%, *p* < 0.01; women, 2.4% vs. 4.9%, *p* < 0.01; alcohol consumption: men, 66.6% vs. 71.9%, *p* < 0.05; women, 28.0% vs. 35.8%, *p* < 0.001) [25]. These findings suggest that, at least with regard to smoking and drinking habits, the participants in the present study might lead healthier lifestyles compared with the general Japanese population, which may introduce a selection bias. This should be considered when generalizing the study results to wider populations.

Third, this study used serum samples that had been stored for 10 years, and no data are available regarding the stability of such samples during long-term frozen storage. Therefore, the possibility that degradation occurred during storage, thereby resulting in lower values than the actual concentrations, cannot be excluded. However, the serum samples were centrifuged immediately after blood collection and stored in a freezer at −80 °C, which is considered to have ensured favorable serum conditions. Moreover, even if degradation occurred, because all samples were stored under the same conditions, the analysis of the association with dietary intake groups should still be valid.

## 5. Conclusions

In conclusion, the results of the present study suggest that blood EGT concentrations in the Japanese population vary by age and gender, and that EGT is positively correlated with not only mushrooms, but also fish and nutrients abundant in fish, such as n-3 fatty acids, vitamin D, and vitamin B_12_. In the future, we plan to apply these findings to follow-up investigations of the ROAD study to examine further how blood EGT concentrations reflect health status.

## Figures and Tables

**Table 1:** Characteristics of the participants in the present study.

	Total	Men	Women	*p*-Value
No. of participants	1457	474	983	-
Age (years)	65.6 ± 13.0	66.4 ± 13.6	65.2 ± 12.7	0.11
Height (cm)	156 ± 9.1	164.7 ± 7.2	151.8 ± 6.7	<0.001
Weight (kg)	55.6 ± 11.2	63.4 ± 11.4	51.8 ± 8.8	<0.001
BMI (kg/m^2^)	22.7 ± 3.5	23.3 ± 3.4	22.5 ± 3.5	<0.001
Serum EGT concentration (μM) Median (IQR)	0.74 (0.39–1.37)	0.67 (0.35–1.26)	0.78 (0.40–1.43)	<0.01

Age, height, weight, and BMI are expressed as mean ± standard deviation and serum EGT concentration is expressed as median (interquartile range; IQR). Nonpaired Student’s *t*-test was used to compare age, height, weight, body mass index, and serum ergothioneine (EGT) concentrations between men and women.

**Table 2:** Correlation between serum ergothioneine (EGT) concentrations and diet.

Diet	Intake Amount (g/Day)	*r*	*p*-Value
Total fish	145.3 ± 101.7	0.215	<0.001
Mushroom	10.6 ± 9.5	0.202	<0.001
Grilled fish	47.5 ± 41.9	0.200	<0.001
Cooked sugar	3.3 ± 1.8	0.173	<0.001
Fish with bones	17.9 ± 22.8	0.168	<0.001
Cooked salt	3.4 ± 1.3	0.157	<0.001
Oily fish	23.2 ± 23.4	0.152	<0.001
Tofu and fried tofu	45.4 ± 36	0.148	<0.001
Fried food	24.5 ± 21.6	−0.132	<0.001
Braised fish	56.6 ± 48.6	0.130	<0.001
Braised food	104.4 ± 66.5	0.128	<0.001
Bread	48.3 ± 29.1	−0.123	<0.001
Seaweed	10.8 ± 11	0.123	<0.001
Green leafy vegetables	34.7 ± 32.9	0.121	<0.001
Dried fish	25 ± 23.5	0.118	<0.001
Ramen noodles	12.7 ± 17.8	−0.091	0.001
Carrots and pumpkins	20.9 ± 17.8	0.091	0.001
Grilled meat	11.1 ± 13.1	−0.088	0.001
Cooking oil	9.4 ± 5.1	−0.078	0.003
Cabbage	39.8 ± 31.9	0.075	0.004
Sake	18.9 ± 63.5	0.068	0.009
Soy sauce	1.6 ± 0.4	−0.067	0.010
Persimmon/Strawberry	28.5 ± 37.6	−0.066	0.012
Sugar	2.8 ± 4.5	−0.064	0.014
Ham	6.8 ± 8.2	−0.064	0.014
Radish/tuber	20.7 ± 20.5	0.062	0.019
Hamburger steak	25.1 ± 22.8	−0.060	0.023
Citrus fruits	49.5 ± 51.5	−0.056	0.032
Beer	69.7 ± 174.7	0.054	0.039
Udon noodles	20.4 ± 22.6	0.051	0.051
100% juice	48.1 ± 86.9	−0.050	0.058
Rice	315.7 ± 166.3	0.048	0.068
Liver	0.8 ± 2.8	0.047	0.075
Ice cream	11.2 ± 23.7	−0.046	0.082
Pickles (other)	12.5 ± 14.1	0.045	0.086
Low-fat fish	16.3 ± 18.5	0.042	0.110
Canned tuna	2.7 ± 5.5	0.041	0.115
Coke	50.6 ± 104.2	−0.041	0.116
Mayonnaise	5.8 ± 5.4	−0.040	0.123
Raw fish	29.4 ± 28.7	−0.040	0.125
Natto	7.0 ± 13.1	0.038	0.149
Japanese sweets	10 ± 12.1	0.036	0.168
Citrus fruits (seasonal)	21.3 ± 15.9	0.035	0.184
Confectionery	20.6 ± 24.5	−0.034	0.190
Potato	49.8 ± 44.8	0.033	0.208
Wine	2.7 ± 18.2	0.032	0.221
Tempura/fried fish	19.8 ± 19.7	−0.032	0.222
Miso soup	129.1 ± 110.8	0.031	0.244
Other	38.7 ± 40.5	−0.030	0.254
Green tea	243.4 ± 232.2	−0.029	0.264
Chicken	23.2 ± 20.1	−0.027	0.295
Coffee	219.1 ± 176.7	0.026	0.329
Strawberries (seasonal)	8 ± 11	0.024	0.350
Pork and beef	27.8 ± 20.4	0.024	0.367
Pickles (green leafy vegetables)	9.1 ± 10.2	0.020	0.434
Milk	74.7 ± 81.5	−0.019	0.466
Tomatoes	22.3 ± 25.8	0.019	0.480
Rice crackers	9.9 ± 12.4	0.017	0.522
Oysters (seasonal)	10.5 ± 11.3	0.015	0.570
Stir-fry	43.7 ± 32.4	0.015	0.573
Noodle soup	60.6 ± 56.6	−0.014	0.586
Whiskey	0.8 ± 8.9	−0.012	0.642
Pasta	9.0 ± 15.5	−0.009	0.739
Squid, octopus, shrimp, shellfish	11.6 ± 13.5	−0.008	0.746
Shochu	12.0 ± 38.3	−0.008	0.768
Tea/oolong tea	48.2 ± 113.1	−0.007	0.777
Soba	9.5 ± 16.9	−0.006	0.822
Eggs	36.6 ± 25.2	−0.003	0.901
Low-fat milk	37.9 ± 68.1	−0.002	0.934
Root vegetables	33.6 ± 26	0.001	0.962

Data are expressed as mean intake amount ± standard deviation. Total fish was calculated by adding together grilled fish, fish with bones, oily fish, braised fish, dried fish, low-fat fish, canned tuna, raw fish, and tempura/fried fish.

**Table 3:** Correlation between serum ergothioneine (EGT) concentrations and dietary nutrients.

Nutrient		Intake Amount		*r*	*p*-Value
Energy, PFC	Energy	1808.2 ± 574.8	kcal/day	0.041	0.12
	Protein	70.1 ± 27.8	g/day	0.109	<0.001
	Animal protein	42 ± 21.7	g/day	0.121	<0.001
	Vegetable protein	28.1 ± 9.2	g/day	0.046	0.081
	Lipids	49.4 ± 19.9	g/day	0.009	0.742
	Animal fat	23.8 ± 11.7	g/day	0.070	0.007
	Vegetable lipids	25.6 ± 10.6	g/day	−0.062	0.018
	Carbohydrates	250.1 ± 81.9	g/day	0.008	0.755
Minerals	Ash	18.6 ± 6.5	g/day	0.107	<0.001
	Sodium	4286.3 ± 1517.4	mg/day	0.104	<0.001
	Potassium	2535.8 ± 1028.9	mg/day	0.079	0.003
	Calcium	567.1 ± 263.6	mg/day	0.118	<0.001
	Magnesium	250.7 ± 95.8	mg/day	0.120	<0.001
	Phosphorus	1078.4 ± 437	mg/day	0.122	<0.001
	Iron	7.5 ± 3.1	mg/day	0.105	<0.001
	Zinc	7.9 ± 2.8	mg/day	0.093	<0.001
	Copper	1.1 ± 0.4	mg/day	0.075	0.004
	Manganese	3.2 ± 1.2	mg/day	0.041	0.12
Vitamins	Retinol	400.1 ± 421.7	μg/day	0.076	0.004
	Retinol equivalent	699.9 ± 508.9	μg/day	0.102	<0.001
	Vitamin D	19.2 ± 14.2	μg/day	0.180	<0.001
	α-Tocopherol	7.2 ± 3	mg/day	0.036	0.168
	Vitamin K	243.1 ± 151.9	μg/day	0.094	<0.001
	Vitamin B_1_	0.8 ± 0.3	mg/day	0.067	0.01
	Vitamin B_2_	1.2 ± 0.5	mg/day	0.069	0.008
	Niacin	17.7 ± 7.6	mg/day	0.131	<0.001
	Vitamin B_6_	1.3 ± 0.5	mg/day	0.102	<0.001
	Vitamin B_12_	11.5 ± 7.7	μg/day	0.155	<0.001
	Folic acid	330.6 ± 145.4	μg/day	0.077	0.003
	Pantothenic acid	6.2 ± 2.4	mg/day	0.066	0.012
	Vitamin C	129.4 ± 70.4	mg/day	−0.012	0.634
Fatty acids	Fatty acids (*n*-3)	2.8 ± 1.4	g/day	0.123	<0.001
	Fatty acids (*n*-6)	9.1 ± 3.7	g/day	−0.024	0.365
	Docosahexaenoic acid	706.9 ± 509.4	mg/day	0.175	<0.001
	Eicosapentaenoic acid	437.4 ± 338.9	mg/day	0.182	<0.001
	Saturated fatty acids	13 ± 5.7	g/day	−0.006	0.826
	Monounsaturated fatty acids	17.5 ± 7.3	g/day	−0.009	0.727
	Polyunsaturated fatty acids	11.9 ± 4.8	g/day	0.019	0.468
Fiber	Soluble dietary fiber	2.8 ± 1.3	g/day	0.022	0.404
	Insoluble fiber	8.4 ± 3.4	g/day	0.054	0.041
	Total dietary fiber	11.6 ± 4.9	g/day	0.052	0.047
Carotenoids	β-Carotene equivalent	3558.5 ± 2356.9	μg/day	0.102	<0.001
Others	Salt equivalent	10.8 ± 3.8	g/day	0.103	<0.001
	Alcohol	8.4 ± 17.4	g/day	0.047	0.071
	Daidzein	10.2 ± 7.9	mg/day	0.122	<0.001
	Genistein	17.3 ± 13.4	mg/day	0.123	<0.001
	Cholesterol	389 ± 191.2	mg/day	0.079	0.003

Data are expressed as mean intake amount ± standard deviation. PFC: protein, fat, carbohydrate.

## Data Availability

The data are not publicly available because of privacy concerns. The data presented in this study are available in this text.

## Supplementary Materials

The following supporting information can be downloaded at: https://www.mdpi.com/article/10.3390/nu17030517/s1, Table S1: Serum ergothioneine (EGT) concentration in each age stratum adjusted by mushroom intake; Table S2: Serum EGT concentration by gender adjusted by total fish and mushroom intake; Table S3: Partial correlation among serum ergothioneine (EGT) concentrations, mushroom intake, and total fish intake.
