# Supplementary material for: The Association Between Serum Ergothioneine Concentration and Japanese Dietary Habits: The Third Survey of the ROAD Study
Source: Nutrients. 2025 Jan 30;17(3):517. doi: 10.3390/nu17030517 (PMC11820927; doi:10.3390/nu17030517)
Supplement: Supplementary file 1 [file nutrients-17-00517-s001.zip › nutrients-3438749-supplementary.pdf]

Table S1. Serum ergothioneine (EGT) concentration in each age stratum adjusted by mushroom intake.

Sex	Age (years)	n	Non-adjusted		Adjusted by mushroom intake	
			Serum EGT concentration	*p*-value vs. ≤ 49 years	Serum EGT concentration	*p*-value vs. ≤ 49 years
Men	≤ 49	53	0.80 ± 0.11	-	0.82 ± 0.14	-
	50s	76	0.92 ± 0.13	0.887	0.92 ± 0.12	0.933
	60s	126	1.99 ± 0.10	0.52	0.99 ± 0.09	0.6387
	70s	134	1.24 ± 0.10	0.03	1.21 ± 0.09	0.0564
	≥ 80	85	0.89 ± 0.08	0.937	0.94 ± 0.11	0.8551
Women	≤ 49	115	0.86 ± 0.09	-	0.85 ± 0.13	-
	50s	180	1.09 ± 0.09	0.411	1.08 ± 0.11	0.4074
	60s	284	1.54 ± 0.12	< 0.001	1.52 ± 0.08	< 0.001
	70s	285	1.17 ± 0.07	0.135	1.18 ± 0.08	0.0988
	≥ 80	119	0.95 ± 0.09	0.953	1.01 ± 0.13	0.7734

Data are expressed as mean ± standard error. Dunnett's test was used to compare between age strata (50–59, 60–69, 70–79 and ≥ 80 years) vs. ≤ 49 years by sex.

Table S2. Serum EGT concentration by gender adjusted by total fish and mushroom intake.

	Non-adjusted			Adjusted by total fish and mushroom intake		
	Men	Women	*p*-value	Men	Women	*p*-value
Serum EGT concentration (μM)	1.01 ± 0.05	1.20 ± 0.05	< 0.01	1.01 ± 0.06	1.20 ± 0.04	< 0.01

Data are expressed as mean ± standard error. Student's t test was used to compare between men and women.

Table S3. Partial correlation among serum ergothioneine (EGT) concentrations, mushroom intake, and total fish intake.

	Serum EGT concentration	Mushroom intake	Total fish intake
Serum EGT concentration		$r = 0.150$ ($p < 0.001$)	$r = 0.168$ ($p < 0.001$)
Mushroom intake			$r = 0.251$ ($p < 0.001$)
Total fish intake			
